# MicroRNA-derived network analysis of differentially methylated genes in schizophrenia, implicating GABA receptor B1 [GABBR1] and protein kinase B [AKT1]

**DOI:** 10.1186/s13062-015-0089-y

**Published:** 2015-10-08

**Authors:** Vadim Gumerov, Hedi Hegyi

**Affiliations:** CEITEC - Central European Institute of Technology, Masaryk University, Kamenice 5, 62500 Brno, Czech Republic

**Keywords:** Schizophrenia, Methylation, GABBR1, AKT1, MicroRNAs, Gene networks, Protein-protein interactions

## Abstract

**Background:**

While hundreds of genes have been implicated already in the etiology of schizophrenia, the exact cause is not known or the disease is considered multigenic in origin. Recent discoveries of new types of RNAs and the gradual elimination of the “junk DNA” hypothesis refocused the attention on the noncoding part of the human genome. Here we re-analyzed a recent dataset of differentially methylated genes from schizophrenic patients and cross-tabulated them with *cis* regulatory and repetitive elements and microRNAs known to be involved in schizophrenia.

**Results:**

We found that the number of schizophrenia-related (SZ) microRNA targets follows a scale-free distribution with several microRNA hubs and that schizophrenia-related microRNAs with shared targets form a small-world network. The top ten microRNAs with the highest number of SZ gene targets regulate approximately 80 % of all microRNA-regulated genes whereas the top two microRNAs regulate 40–52 % of all such genes. We also found that genes that are regulated by the same microRNAs tend to have more protein-protein interactions than randomly selected schizophrenia genes. This highlights the role microRNAs possibly play in coordinating the abundance of interacting proteins, an important function that has not been sufficiently explored before. The analysis revealed that GABBR1 is regulated by both of the top two microRNAs and acts as a hub by interacting with many schizophrenia-related genes and sharing several types of transcription-binding sites with its interactors. We also found that differentially methylated repetitive elements are significantly more methylated in schizophrenia, pointing out their potential role in the disease.

**Conclusions:**

We find that GABBR1 has a central importance in schizophrenia, even if no direct cause and effect have been shown for it for the time. In addition to being a hub in microRNA-derived regulatory pathways and protein-protein interactions, its centrality is also supported by the high number of *cis* regulatory elements and transcription factor-binding sites that regulate its transcription. These findings are in line with several genome-wide association studies that repeatedly find the major histocompatibility region (where GABBR1 is located) to have the highest number of single nucleotide polymorphisms in schizophrenics. Our model also offers an explanation for the downregulation of protein kinase B, another consistent finding in schizophrenic patients. Our observations support the notion that microRNAs fine-tune the amount of proteins acting in the same biological pathways in schizophrenia, giving further support to the emerging theory of competing endogenous RNAs.

**Open Peer Review:**

The manuscript was reviewed by Jaap Heringa, Sandor Pongor and Zoltan Gaspari.

**Electronic supplementary material:**

The online version of this article (doi:10.1186/s13062-015-0089-y) contains supplementary material, which is available to authorized users.

## Background

Schizophrenia, a devastating disease affecting about 1 % of the human population, has been first described and phenomenologically characterized for the English-speaking world by Emil Kraepelin in 1919, nearly a hundred years ago [1]. Nevertheless, the exact cause of the disease is still not known and there are no molecular tests that would help diagnose a new case. While it is possible to identify the phenotype of the disease in most cases, with a clear genetic component, the scientific community is settling on the consensus that it is a polygenic disease [2, 3] with many different individual genes contributing to it, with distinct mutations in different families and geographic regions.

The recently discovered altered microRNA expression in schizophrenia [4] gave rise to new theories regarding the cause of schizophrenia, highlighting the importance of gene networks [5], further supporting the notion that schizophrenia is a multigenic disease.

In this work we analyzed several datasets, namely: a genome-wide data set of differentially methylated CpGs in a large cohort of schizophrenic patients using Illumina’s 450 k probe set [6], all known microRNAs known to be differentially expressed in schizophrenia and compared them with a set of genes annotated by Genecards [7] to be related to schizophrenia and related them to known protein-protein interactions as recorded in STRING [8].

We found that the targets of microRNAs follow a power-law distribution where most miRNAs have a small number of gene targets and a few miRNAs have many. The two miRNAs with the highest number of targets both regulate GABBR1, a feature shared only by a few other schizophrenia-related genes. GABBR1 had also the highest number of differentially methylated CpGs in a subset of patients (in [6]), all of them hypermethylated whereas a large majority of the differentially methylated genes had both hyper- and hypomethylated CpGs in the same study.

A protein-protein interaction network, constructed from schizophrenia-related, differentially methylated genes, regulated by one of the top two microRNAs revealed that almost all of the interacting partners of GABBR1 were also hypermethylated. AKT1, a protein kinase B, known to be downregulated in schizophrenia, is also regulated by miR-26b, the second most abundant microRNA, and both proteins interact with ATF2, activating transcription factor 2, which is also hypermethylated in schizophrenics in [6].

Our analysis supports a scenario where the initial pathogenic event might be the downregulation of GABBR1 (possibly brought about by an external factor such as a viral infection as we hypothesized before in [9]) perhaps through one of the regulatory cis elements of GABBR1, whose perturbation is propagated into its network of genes, connected via shared microRNAs.

We also found that CpG probes that match repetitive elements in the human genome are significantly hypermethylated in schizophrenics.

## Methods

### Data collection

We collected schizophrenia-related microRNAs from the literature [10–26] and retrieved their experimentally validated targets from miRTarBase [27]. Schizophrenia related genes were retrieved from Genecards, v. 3.12 [7] and Malacards [28]. Differentially methylated genes were taken from Wockner et al. [7], miRNA abundances were taken from miRBase (release 21) [29].

### Power law fitting and testing

We cross-tabulated the genes from the 3 datasets: experimentally validated targets of miRNAs implicated in schizophrenia, genes related to schizophrenia and genes differentially methylated in schizophrenia. We then counted the number of target genes for each miRNA in this combined dataset and fitted a power law distribution to the resulting counts.

Goodness of fit test was performed using Kolmogorov-Smirnov statistic and bootstrapping procedure as described in [30] using the “poweRlaw” package [31] in R. We used 2500 synthetic sets which allowed *p*-value to be accurate to about 2 decimal digits [30]. Here *p*-value is the fraction of the time the resulting Kolmogorov-Smirnov statistic for the synthetic set is larger than the statistic for the empirical data. Thus small *p*-values allow us to rule out power law distribution. In our test we obtained *p*-value = 0.25. Considering *p*-value = 0.1 as a threshold [30] we cannot rule out power law distribution for this subset of the data.

### Small-world net

We constructed a miRNA-miRNA network based on shared targets. To see if the network has small-world property, we constructed random graphs using the Erdős–Rényi algorithm with the same number of nodes and the same average number of edges for each node. We compared the clustering coefficients and characteristic path lengths between the two graphs. The clustering coefficient proved to be bigger for the real graph than the random one while the characteristic path length were the same – thus meeting the criteria for small world property [32].

### Protein-protein interaction network construction

Protein-protein interaction networks of miRNA targets were constructed based on the interactions data in STRING v9.1 [8]. Combined score cutoff used to include an interaction was 0.4. The network for the top 2 miRNAs was visualized using Cytoscape, v.3.2.0 [33]. Differences of M-values between schizophrenia patients and healthy subjects for genes are averages of differences for probes reported differentially methylated in [6].

### Interactions between genes regulated by the same miRNA

We selected genes randomly from the list of all schizophrenia-related genes in the amount equal to the number of genes regulated by each miRNA and counted the number of interactions between them. Calculated interactions were normalized by dividing them by the maximum number of edges in the corresponding undirected graph for each miRNA target set. This procedure was repeated 3000 times. We subtracted from the normalized number of interactions between genes regulated by the same miRNA the number of interactions (normalized) in each corresponding simulated gene set. This was done for all miRNAs implicated in schizophrenia and for the results single-sample Wilcoxon signed-rank tests were performed.

### Differentially methylated repetitive elements

As explained in [6] the authors organized their differentially methylated CpG probes into 3 separate data sets (in three supplementary tables in [6]), where set 1 contained all the differentially methylated probes between patients and controls, set 2 contained the age-adjusted values, whereas set 3 contained differentially methylated probes between two markedly different patient subgroups.

After acquiring the sequences of the Illumina 450 k probe set (120 nucleotides each, surrounding the middle CpG dinucleotide queried in the methylation studies) we determined if they are substantially similar to any repetitive elements. To do this we ran BLASTN [34] to compare the sequences of all probes to the human subset of Repbase [35], a public database of all repetitive elements in the human genome. We then selected those probes that match a repetitive element using an e-value threshold of 1e-5, for each probe selecting the best matching repetitive element. We then sorted the Mval values (the measure of differential methylation in [6] between schizophrenics and controls or between the two subgroups of patients) for all differentially methylated probes and also the subset of repetitive probes into 21 bins (in the range from −1 to +1, with increments of 0.1) for each of the 3 sets of differentially methylated probes in [6] and counted the number of probes in each bin. At the end each distribution was normalized to 100 as shown in Fig. 5, so that adding up the values amounts to 100 in each case.

To determine if the values differ between the entire sets and those containing only the repetitive elements for each of the 3 data sets in [6] we used Student’s *t*-test. We repeated the tests by separating the negative and positive values to avoid unnecessary complexities arising from the bimodal character of the methylation value distributions.

### Code availability

All the analyses, data randomizations and statistical tests were carried out by in-house Perl, Python and R scripts. They are freely available from the authors on request.No experiments were carried out on any living creatures.

## Results

### Cross-tabulation of microRNAs, schizophrenia genes and differentially methylated genes in schizophrenia

First we collected all schizophrenia-related microRNAs from the literature [10–26]. We then cross-tabulated (for the overlap between the various data sets see Additional file 1: Figure S1) this set of 129 microRNAs with those genes that were differentially methylated in schizophrenics according to [6] (using subset 2 from [6] where the differentially methylated probes were corrected for age and PMI, post-mortem interval), and are regulated by any of the 129 microRNAs according to mirTarBase [27], a collection of experimentally verified targets of microRNAs (shown in Additional file 2: Table S1). We then took a list of 2065 genes associated with schizophrenia according to Genecards [7] and cross-tabulated them with the differentially methylated set of genes from [6], selecting only those genes that are also regulated by at least one microRNA in mirTarBase.

Table 1 shows the 253 genes associated with schizophrenia in Genecards [7] and regulated by a microRNA, ranking the microRNAs according to the number of their known targets. Table 1 shows only the top ten microRNAs, the latter ranked according to the number of known targets for them. Apparently, out of the 253 schizophrenia-related, microRNA-regulated, differentially methylated genes (in 6) listed by Genecards, 205 (81 %) are regulated by one of the top ten microRNAs (Table 1). However, not every gene is regulated by microRNAs. Out of the 2931 genes differentially methylated in schizophrenics in [6] only 1547 (53 %) are regulated by at least one microRNA implicated in schizophrenia (Additional file 2: Table S1). This is in line with the finding of others, who also found that in schizophrenia only about half of all coding genes are regulated by a microRNA [36].

**Table 1:**
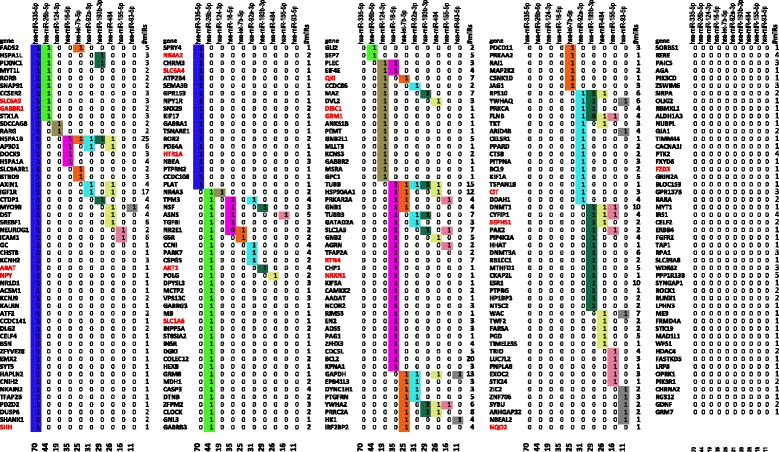
Differentially methylated genes associated with schizophrenia according to Genecards targeted by schizophrenia-related microRNAs, cross-tabulated for the top ten microRNAs. Genes classified as schizophrenia-related by Malacards are shown in red

**Fig. 1:**
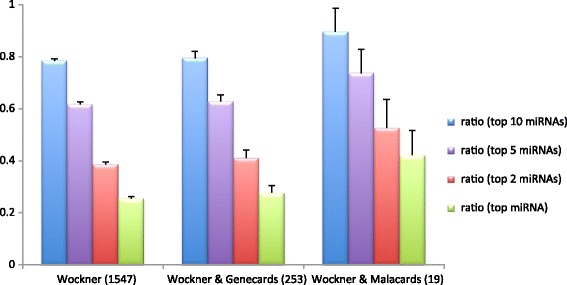
The ratio of genes regulated by the top ten (blue), top five (purple) or top two (red) microRNAs and by the top microRNA (green), respectively, for various data sets. The Wockner data set refers to data set 2 in [6] that are combined with genes listed in Genecards and Malacards, respectively. The error bars show standard deviations acquired via random selection of 155 (1/10^th^ of all genes), 253 (overlapping genes with Genecards) or 19 genes (overlap with Malacards), respectively (repeated 1000 times for each value), each time calculating the ratio of genes that are regulated by the top 10, 5, 2 or 1 microRNAs

We also indicated (gene names typed in red in Table 1) if the gene is associated with schizophrenia according to Malacards [28], a compendium of diseases and related genes. In Fig. 1 the ratio of microRNA-regulated genes are shown for various numbers of microRNAs (for the top, top two and top ten microRNAs, respectively; see also Additional file 3: Figure S2), across several gene sets, such as all the differentially methylated genes in [6] (we called the Wockner set, after the first author of that paper) those in Genecards [7] and Malacards [28]. Remarkably, approximately 80 % of all miRNA-regulated, schizophrenia-related genes are targeted by only the ten most frequently occurring microRNAs, a ratio that increases to 89 % when we consider only the Malacards genes (17 out of 19 genes, shown in red in Table 1). Regarding the top five, top two and the single top microRNA with the most targets, there is also a tendency for their relative number of targets to increase when the schizophrenia-related evidence increases. The ratio of the targets of the top two microRNAs is around 40 % for the first data set (where all differentially methylated genes from Wockner et al. [6] are taken into account), however it increases to 46 % and 53 %, for Genecards and Malacards, respectively. Figure 1 shows that in all four cases the ratios for the targets increases with increasing evidence for schizophrenia (i.e. always the highest for the Malacards set). This may support the role of microRNAs in schizophrenia in general and that of the top two microRNAs in particular.

**Fig. 2:**
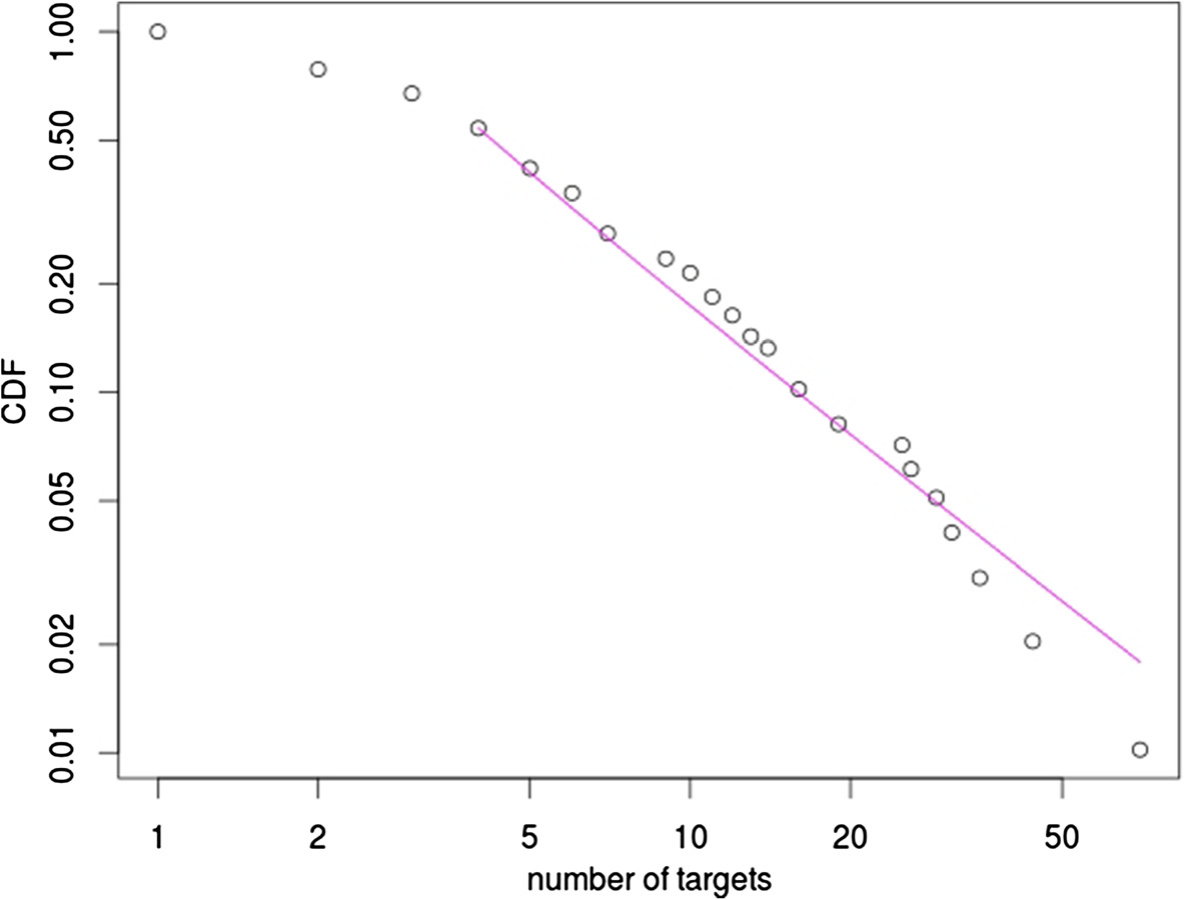
Cumulative Distribution Function (CDF) of the number of targets of microRNAs in schizophrenia shown as the fraction of the total number of targets on a log-log scale. Only genes found differentially methylated by Wockner et al. [6] are taken into account. The distribution could be fitted with a straight line, which is typical of scale-free networks, i.e. the fraction of nodes having k connections in the network could be described by P(k) ~ k^-c^ where **c** is a constant exponent, characterizing the distribution. For this network **c** was calculated to be 2.14

Using the known miRNA-target relations we constructed two types of networks: a miRNA- > target and a miRNA-miRNA network. The network of miRNAs and the genes regulated by them, not including interactions between these genes, is a bipartite directed network with miRNA- > target directed links. This network can be characterized by in-degree (in this case the number of miRNAs controlling a given gene) distribution and out-degree (the number of genes controlled by a given miRNA) distribution. In Fig. 2 we depicted the out-degree distribution of this network. We tested goodness of fit using Kolmogorov-Smirnov statistic as described in Methods, which demonstrated that this distribution follows the power law.

Analysis of the miRNA-miRNA network constructed based on the shared genes between any two miRNAs demonstrated small-world properties. By definition [32], the clustering coefficient of a small world net is bigger than the clustering coefficient of a random graph (with the same number of nodes and same average number of edges per node), while characteristic path lengths are approximately equal [32]. Clustering coefficient and characteristic path length of our network are 0.807 and 1.717, respectively, while these parameters for the corresponding random network are 0.330 and 1.669, respectively.

### Top two microRNAs and protein-protein interactions among schizophrenia-related genes

To investigate the potential role microRNAs might play in coordinating protein-protein interactions (PPIs), using STRING [8] we determined the number of PPIs for the network of schizophrenia-related proteins regulated by the two most frequently occurring microRNAs, mir-335 and mir-26b. To see if they have more interactions than those proteins not regulated by the same microRNAs we performed randomization and a statistical test described in the Methods section. Results are shown in Additional file 4: Table S3. Apparently, proteins that are regulated by the same microRNAs tend to have more interactions and one of the main regulatory roles of the microRNAs might be actually this coordinating effect, to make sure that interacting proteins have the correct stoichiometry in the cell [37].

**Fig. 3:**
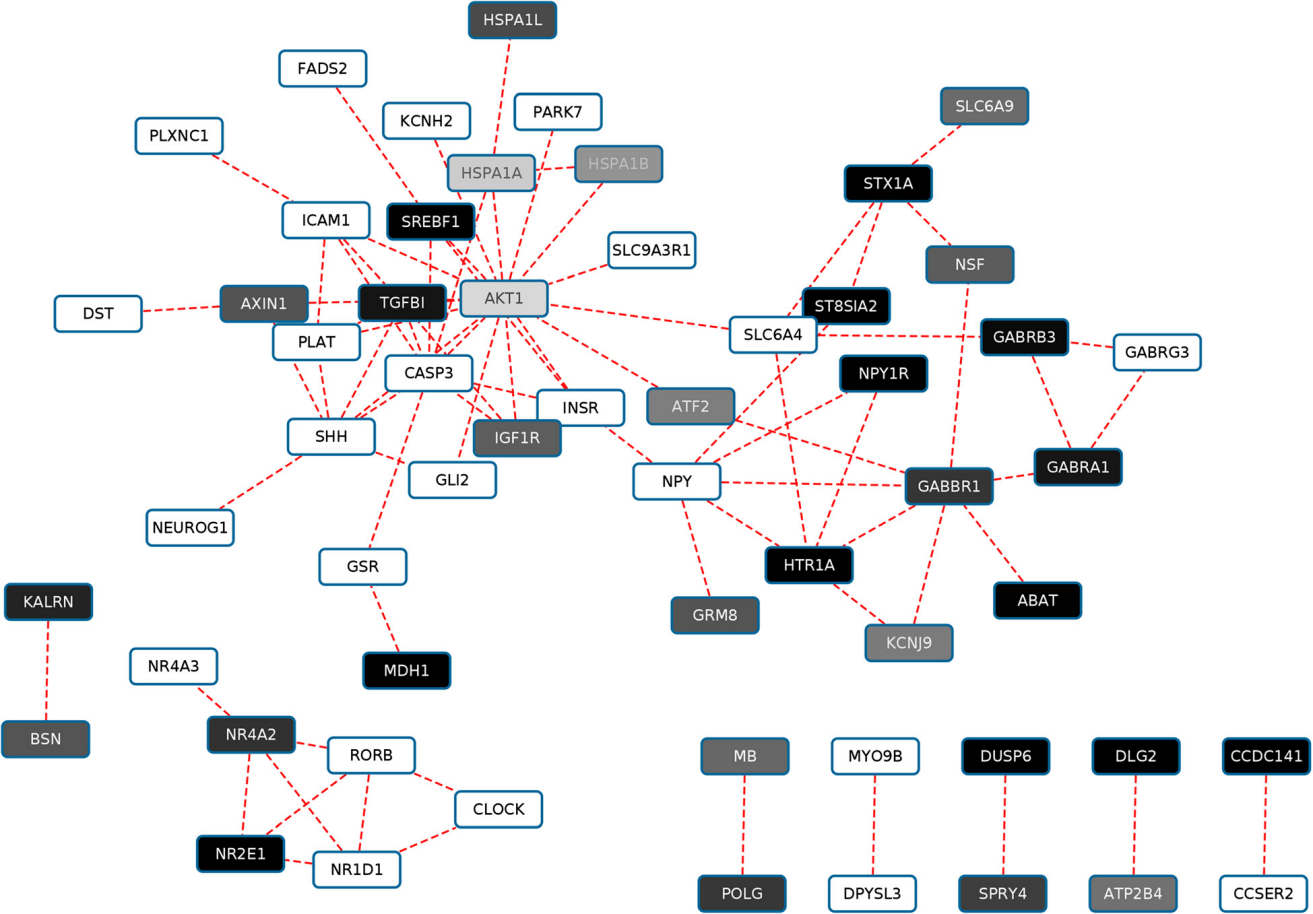
Protein targets of mir-335 and mir-26b and their known interactions according to STRING v9.1 [8], constructed by Cytoscape. The shading of each symbol reflects the average of the M values reported differentially methylated between schizophrenia patients and controls

Protein-coding genes that interact with each other according to STRING, are associated with schizophrenia and regulated by mir-335 or mir-26b, are shown in Fig. 3. The network contains two main hubs, AKT1 and GABBR1, which, although do not have a direct interaction, both interact with ATF2. AKT1, protein kinase B is an important molecule in signaling pathways and is known to be downregulated in recent-onset schizophrenia although the source of this downregulation is not known [38]. While the interacting partners of AKT1 are both hyper- and hypomethylated (the level of methylation indicated on a grey scale for each partner, the most methylated genes shown in black) the interactors of GABBR1 are almost all hypermethylated, with the exception of NPY, neuropeptide Y. GABBR1 was also the most consistently hypermethylated gene in the entire set in [6].

**Fig 4:**
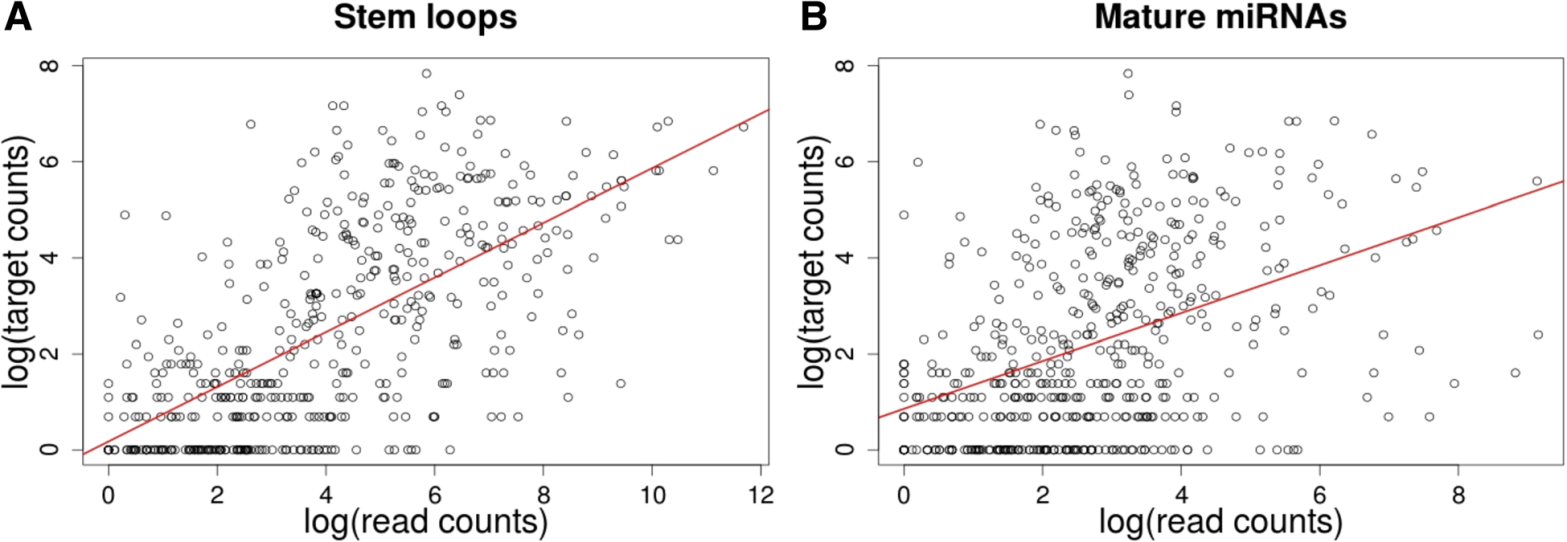
Scatterplot of the number of experimentally validated targets (taken from mirTarBase) vs. the abundance of miRNAs averaged across several tissues (taken from mirbase.org). **a**. miRNA stem-loop transcripts read counts; **b**. mature miRNA read counts

We also checked the abundance of the microRNAs taken from the resource mirbase.org, which has the most comprehensive annotation about microRNAs in human tissues across tens of different experiments [29]. After performing Shapiro-Wilk normality test, which indicated non-normal distribution of the data, we have run Spearman’s rank correlation test. Using those human miRNAs that have experimentally validated targets in miRTarBase, we obtained statistically significant positive correlation between target counts and mature miRNA read counts (rho = 0.44, *p*-value < 2.2e-16) (Fig. 4a) as well as between target counts and stem-loop transcripts read counts (rho = 0.67, *p*-value < 2.2e-16) (Fig. 4b). Statistically significant positive correlation was also observed when we use only the schizophrenia related miRNA set (Additional file 5: Figure S3). Thus, these results support the theory of “competing endogenous RNAs” [39], which inherently assumes that microRNAs with more targets are also expressed in higher quantities, to carry out their regulatory functions.

### *Cis* regulatory elements for schizophrenia-related genes

To see if regulation of gene expression by microRNAs can be related to *cis* regulatory elements (enhancers and silencers) regulating the transcription of schizophrenia-related genes we also listed the number of *cis* and promoter elements identified by Thurman et al. [40] for each gene that was present in their study. The numbers for both elements are listed in Additional file 2: Table S1. A correlation analysis showed that the number of regulating microRNAs or the presence of certain microRNAs does not correlate with the numbers of promoter and *cis* elements (data not shown), thus apparently providing independent regulatory mechanisms, regulating different aspects of gene expression.

### Transcription factor binding sites in cis regulatory elements of GABBR1 and 16 network-neighborhood genes

We also studied transcription factor binding sites (TFBSs) in the regulatory *cis* and promoter elements of GABBR1 and 16 of its neighboring genes in the PPI network in Fig. 3 to see if the interacting genes share any common TFBS motifs. We mapped the motifs identified for the entire human genome by [41] to the regulatory elements of GABBR1 and 16 of its neighbors in the PPI map in Fig. 3, the results are shown in Additional file 6: Table S2. Apparently, the table is highly populated, with the most highly represented transcription factor binding sites appearing more than 20 times. GABBR1 shares several TFBS motifs with most of the interacting proteins (at least one with each) and neighborhood proteins in Fig. 3.

### Differential methylation of repetitive elements in schizophrenic brains

We also investigated the potential role repetitive elements might have in the etiology of schizophrenia, with special respect to endogenous retroviruses that have been found by several studies to be abnormally expressed at the onset of the disease [42]. We hypothesized before [9] that a long terminal repeat (LTR) of the endogenous retrovirus HERV-W in the *cis* regulatory region of GABBR1 might account for the downregulation of GABBR1 in schizophrenia [43].

We studied the methylation of the repetitive elements by comparing the sequences of the differentially methylated probes in all three data sets in [6] to Repbase, a collection of all repetitive elements in eukaryotic genomes [35] using BLASTN as described in Methods.

**Fig. 5:**
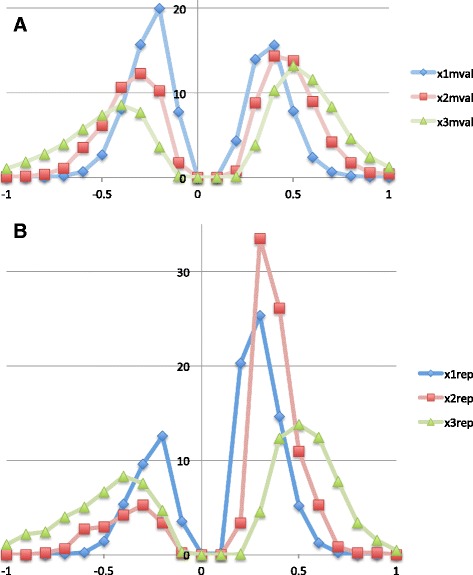
Methylation value distributions for CpG probes in repetitive elements. **a**. All differentially methylated probes for the 3 data sets in [6]. **b**. Probes in the 3 data sets overlapping a repetitive element in RepBase [35]

While we did not find any particular repetitive element (such as the LTR of an endogenous retrovirus, e.g. HERV-W) enriched in the differentially methylated probe sequences (each probe sequence defined as 121 nucleotides, encompassing the queried CpG dinucleotide in the middle), using Student’s *t*-test we did find data sets 1 and 2 significantly more methylated (*p*-value < 1e-5) if they matched a repetitive element. It can be observed in Fig. 5 by comparing the histograms of methylation values (x1_mval through x3_mval, where x1, x2 and x3 refer to the 3 differentially methylated sets of probes in [6]) derived from the entire sets (Fig. 5a) to methylation values derived from a subset of probes in each respective set (x1 to x3) that matched a repetitive element in Repbase (x1rep, x2rep and x3rep in Fig. 5b), as described in Methods. Apparently, for the x1 and x2 sets (that represent differentially methylated probes between patients and controls, with x2 containing only age- and HMI-corrected probes of x1) the repetitive probes (x1rep and x2rep in Fig. 5b) have significantly higher numbers of hypermethylated probes (higher values in the positive range in Fig. 5b) when compared to the distributions in Fig. 5a. For the x3 data set (representing differentially methylated probes between two distinct patient groups) there was only a marginal difference, with the repetitive element matching probes being slightly less methylated than the entire set (*p*-value = 0.017).

## Discussion

Converging experimental and clinical evidence suggests that dysfunction of proper GABAergic inhibition (GABA, gamma-aminobutyric acid is the main inhibitory neurotransmitter in the mammalian brain) in GABA neurons in the cerebral cortex underlies at least part of the pathophysiological process in schizophrenia [44]. In line with the evidence, several pathways and the dysfunction of associated genes were suggested to be the primary cause of schizophrenia, such as that of GAD1 (glutamic acid decarboxylase 1, producing GABA from glutamate) [45], N-methyl-D-aspartate (NMDA) receptor (a glutamate receptor and ion channel protein) and both GABA A [46] and GABA B [9, 43] receptors.

It must be noted that implicating GABA receptors in a GABAergic dysfunction is quite a plausible argument in itself. In this paper we focused on the role GABA B1 receptor (GABBR1) might play in schizophrenia. GABBR1 was recently found to be the most consistently hypermethylated gene in a large-scale methylome study in [6]. GABBR1 is also located in the MHC region that was found to carry the highest number of SNPs in a meta-study of large numbers of schizophrenic patients [3].

In addition, we found that the top two microRNAs with the highest number of targets known to be associated with schizophrenia both regulate GABBR1, a feature shared only by 9 other differentially methylated genes listed in Genecards (Table 1). What is more, the top microRNA in the set, miR-335, regulates 394 of the 1547 differentially methylated genes (25 %) (Additional file 2: Table S1) whereas it regulates 70 of the 253 (28 %) Genecards genes and 8 of the 19 Malacards genes (42 %) (Table 1). There is a similar increase for the fraction of regulated genes for the top 2, top 5 and also for the top ten microRNAs when we limit the number of genes to those of increasing evidence for their involvement in schizophrenia (Fig. 1). What is more, the transcription of GABBR1 is also regulated by a high number of *cis* regulatory and promoter elements (105 and 8, respectively, Additional file 2: Table S1, taken from [40]), highlighting another aspect of central importance of GABBR1.

While several studies have tackled already microRNA-derived networks of interacting proteins both in physiological and disease processes [13, 47, 48], they usually combine both experimental and predicted targets, whereas we found the latter highly non-reliable, depending on the actual prediction method, therefore we restricted our study to experimentally verified microRNA targets only.

We found that the distribution of microRNA targets follows the power law. The miRNA-miRNA network is tightly connected, forming a small-world type of network where the average number of hops needed to reach a node from another is only 1.7. This is also reflected in the fact that the top ten microRNAs regulate 80 % of all microRNA-regulated genes implicated in schizophrenia, a number that increases to 89 % when considering only the cross-section of differentially methylated genes and those identified by Malacards as schizophrenia-related.

After constructing a protein-protein interaction network among the genes that are regulated by either of the top two microRNAs we identified two hubs (Fig. 3), GABBR1 and AKT1, interacting with a high number of other proteins in the network. GABBR1 interacts with GABA receptor A1 and shares network neighbors with several other GABA receptors. Most GABA receptors and the majority of directly interacting proteins, such as HTR1A, a serotonin receptor, are also significantly hypermethylated in the study.

AKT1 has been found to be downregulated in schizophrenia and its downregulation is thought to be instrumental in abnormal hippocampal neuroplasticity and cognition in schizophrenia [49]. While the cause of this downregulation has not been known till recently [38], a recent paper found that the activation of GABA B receptors inhibits Akt1/GSK-3 signaling [50]. AKT1 and GABA B receptors do not directly interact with each other, but via beta-arrestin according to the model in [50]. This model could also explain how perturbing the expression of GABBR1 would change the expression of AKT1 and cause the deregulation of the AKT1-related pathways [51]. While not directly interacting with each other, both AKT1 and GABBR1 interact with ATF2 in our model (Fig. 3), which is also differentially methylated in [6].

Several other bioinformatics studies have reconstructed microRNA-derived networks, including PPIs, protein expression values and transcription factor binding sites for both schizophrenia and other diseases [13, 48, 52], identifying network hubs that have the most potential to influence the networks’ overall behavior. Interestingly, Hansen et al. [13] also identified ATF2 in their network of schizophrenia-related genes although they started out from two other microRNAs, mir-206 and mir-198, in which they found SNPs in the studied schizophrenia patient cohort.

We reconstructed a PPI network for the targets of the top two microRNAs, based on the observation that common miRNA targets tend to appear in the same biochemical pathways (as they tend to have more protein-protein interactions as shown for the top ten microRNAs in Additional file 4: Table S3). As noted by Liang [53], miRNAs have a greater importance in connecting inter-modular than intramodular PPI hubs (i.e. hubs appearing in different vs. the same subnetworks), which also seems to be the case for GABBR1 and AKT1 in our network in Fig. 3. As GABBR1 is regulated by both of the two most prevalent microRNAs, it has an even higher chance to be the major driver of the affected biochemical pathways in schizophrenia.

Genome-wide association studies ranked the extended MHC region (where GABBR1 is also located) to have the highest number of SNPs [3] but this region is also the second most gene-dense genomic region, therefore identifying the exact causative variation will be a very complex task. While no systematic sequencing has been carried out to analyze all mutations in the high number of regulatory elements of GABBR1, the possibility that epigenetic, rather than genetic changes in its vicinity are to account for the disease cannot be excluded either.

## Conclusions

Taken together, we find that GABBR1 has a central importance in schizophrenia, even if no direct cause and effect have been shown for it for the time. The protein-protein interaction map of genes regulated by one of the two top microRNAs shows that GABBR1 and AKT1 are both hub proteins, forming a plausible network of interacting proteins, comprising several key players in the GABA and signaling pathways in schizophrenia. Our model also offers an explanation for the consistent downregulation of AKT1 in recent-onset schizophrenia.

We also found that targets of the same microRNAs tend to have more protein-protein interactions than randomly chosen genes and that microRNA abundance correlates positively with the number of its targets. Both these observations support the notion that microRNAs fine-tune the amount of proteins acting in the same biological pathways in schizophrenia, giving further support to the emerging theory of competing endogenous RNAs [54].

## Reviewers’ comments

### Responses to Reviewer 1 (Jaap Heringa)

We appreciate the reviewer’s efforts and thorough criticism in reviewing our manuscript.

*The authors come up with a few potentially interesting findings on regulation and PPI in schizophrenia, these work more like snapshots than elaborately researched aspects. Furthermore, none of the findings are placed in a biological perspective. For example, if true, why would schizophrenia be more regulated via miRNA than other diseases or normal cell situations?*

We do not say that schizophrenia is “more regulated” by miRNA than other diseases or normal situation, only that miRNA are implicated in schizophrenia: there are several papers some of which we cite that point out the involvement of microRNAs in schizophrenia.

*Stoichiometry of interacting target proteins seems not to be an argument that would hold only for schizophrenia.*

Certainly true but we studied here only schizophrenia-related phenomena/abnormalities.

*I have the following reservations against this manuscript that I think the authors should address:*

*1. As a first point: Somewhat annoyingly, the authors did not insert page numbers in their manuscript, while their line numbering is of no help since line numbers start at 1 at every new page.*

We are sorry about that, we number the pages now.

*2. Although the paper is written compactly, it was at times difficult to grasp what exactly the authors have done. For example, the authors present data on a number of data sets, but it remains unclear how they have assembled these. There are descriptions of data collection in Methods and results, but these appear inconsistent. The authors should improve clarity here.*

We tried to clarify now these issues, referring back to the appropriate Methods section.

*Figure*1*provides some information, but the main text should provide the information. Later on in the paper, the authors talk about “data sets 1 and 2”, where some more explanation would be helpful.*

Data sets 1, 2 and 3 as explained in the text now, refer to the 3 supplementary tables in the large-scale methylome studies by Wockner et al. (reference 6 in our manuscript) where they listed [1] all differentially methylated probes between patients and controls; [2] all differentially methylated probes between patients and controls corrected for age and PMI, post-mortem interval and [3] differentially methylated probes between two patient subgroups, respectively.

*3. Considering the alluded bias for miRNA-directed regulation in schizophrenia, the authors write: “Remarkably, approximately 80 % of all schizophrenia-related genes are targeted by only the ten most frequently occurring microRNAs. We also calculated this ratio for the total number of genes regulated by this set of microRNAs, where it also proved to be a value close to 80 %.” Assuming that “the total number of genes” are all the genes in the Wockner et al. dataset, these statements appear to imply that if there is anything remarkable about the 80 %, it has nothing to do with schizophrenia, as there is no difference between schizophrenia and the general situation in the human brain in this regard. 4. In the text, the authors say that 1547 out of 2931 (53 %) of the genes implicated in schizophrenia (differential methylation – Wockner et al.) are regulated by one or more miRNAs. However, in Fig.*1*the percentages for only the top-10 and top-2 miRNAs (Wockner) are close to 80 and 40, respectively. Something must be wrong here.*

Great point, thanks for pointing this out! As mentioned in the text now, not all schizophrenia-related genes are regulated by microRNAs (as per current knowledge, August 2015, this ratio might change as it is such an actively researched topic). According to others (see Caputo et al., ref 36 in the manuscript), only half of all protein-coding genes are regulated by microRNAs.

*5. The authors make a point on the regulatory control of the top-2 miRNAs by writing: “As shown in Fig.*1*, the ratio of the targets of the top two microRNAs is around 40 % for the first data set (where all differentially methylated genes from Wockner et al.* [6] *are taken into account), however it increases to 46.4 % and 52.6 %, respectively, for the last two data set combinations, which combines the schizophrenia evidence from Genecards and Malacards, respectively. This may support the role of microRNAs in schizophrenia in general and that of the top two microRNAs in particular.” The conclusion that more confirmed data is more correlated with top-2 miRNA-directed regulation is just based on a single dataset of 19 elements. This is not a compelling argument.*

There are 3 data sets: the Wockner set, the Genecards set and the Malacards set. We also added the ratio of targets for the top microRNA, miR-335-5p and also for the top five (in Fig. 1). While there is a more dramatic increase in ratios between the Genecards set and the Malacards set, there is also a slight increase between the Wockner set and the Genecards set. We hope the figure looks more convincing now.

*6. Why did the authors only present data on the top 10 and top 2 miRNAs? They could draw a plot from top 1 to top 15 to show how the coverage falls off.*

We extended Fig. 1 now, also calculating the ratios for the top 5 and the top one microRNAs. We hope the tendency is clear and convincing.

*7. To check the scale free network property, the authors have made a network of miRNAs, but it is unclear to this reviewer what are the nodes and edges in the network. They use miRNAs and the numbers of genes each of those regulate, but how these data are converted in a network is obscure. An edge might have been declared whenever two miRNAs regulate the same target gene, but the authors should be clear on this. If the latter is indeed the procedure that is followed by the authors, then the biological importance of this network is questionable, and so are the findings that this network has scale free and small world properties. - In any event, the fashion of deriving scale free and small world properties is now mainly moot in the biological literature, since this has hardly led to increased biological understanding. As to inferring a power law from double-logarithmic plots, it is quite difficult in practice not to get a straight line using these, so the scale-freeness is not compelling, at least not to this reviewer.*

Thank you for pointing out this lack of clarity in the text. We fixed this in the new version of the paper.

The network consists of miRNAs and genes regulated by them. If we do not include the protein-protein interactions between these genes it can be considered a bipartite directed network (biological example - Martinez and Walhout, 2009 *) with miRNA- > target directed links. This network can be characterized by in-degree (in this case the number of miRNAs controlling a given gene) distribution and out-degree (the number of genes controlled by a given miRNA) distribution. In Fig. 2 we depicted the out-degree distribution of this network. In addition to the plot, we performed goodness of fit test using Kolmogorov-Smirnov statistic as described in the Methods, which demonstrated that this distribution follows the power law.

*Martinez NJ, Walhout AJ. The interplay between transcription factors and microRNAs in genome-scale regulatory networks. Bioessays. 2009 Apr;31 [4]:435–45.

We also constructed a miRNA-miRNA network based on shared genes between any two miRNAs. For this network we found that it has small-world property. Small-world network has high average clustering coefficient and small characteristic path length.

Regarding the biological meaning: Clustering coefficient demonstrates how tightly connected any miRNA is to its neighbors in the miRNA-miRNA network; thus high clustering coefficient can mean that a gene is typically regulated by several microRNAs which can indicate the robustness of this control – malfunction of one miRNA or even several of them can be overpassed by the remaining miRNAs.

Our graph parameters:

Clustering coefficient: 0.807

Characteristic path length: 1.717

After generating a random graph using the Erdos-Renyi algorithm with the same number of nodes and the same average number of links per node (52.327) we found:

Clustering coefficient: 0.330

Characteristic path length: 1.669

Apparently, the real network has a higher clustering coefficient (0.807) than the random Erdos-Renyi graph (0.330).

*8. On the potential role of miRNAs in schizophrenia, the authors write: “To see if they have significantly more interactions than those proteins not regulated by the same microRNAs we performed randomization and a statistical test described in the**Methods**section. Apparently, proteins that are regulated by the same microRNAs tend to have more interactions and one of the main regulatory roles of the microRNAs might be actually this coordinating effect, to make sure that interacting proteins have the correct stoichiometry in the cell* [37]*.” Could the authors provide some data to show that proteins regulated by the same miRNA do indeed interact more?*

We provided the results of simulation in Additional file 4: Table S3 for the top 10 schizophrenia-related miRNAs. In the table the actual connections between targets regulated by the same miRNAs and the average number of connections across 3000 simulations for each miRNA are given.

*9. In addition to a bias in interaction for proteins regulated by the same miRNA, did the authors check whether PPI in schizophrenia is biased in general, so regardless of miRNA regulation?*

Perhaps the referee meant if there are more PPI among the schizophrenia-related genes than between schizophrenia-related and unrelated genes? We did not check this. Considering that there are more than schizophrenia-related 2000 genes, the results would be probably inconclusive.

*10. On miRNA expression, the authors write: “We also checked the abundance of the microRNAs taken from the resource mirbase.org, which has the most comprehensive annotation about microRNAs in human tissues across tens of different experiments* [29]*. The data for the most abundant microRNAs are shown in Fig.*4*, plotted against the number of known targets. Apparently there is a positive correlation between the two values, supporting the theory of “competing endogenous RNAs”* [39]*, which inherently assumes that microRNAs with more targets are also expressed in higher quantities, to carry out their regulatory functions.” Although the authors claim there is a positive correlation (what is the r-value?) the plot looks rather erratic. It would be interesting to check the correlation when some outliers are removed. As another point, was the data used here specifically for schizophrenia? The resource mirbase.org seems more general.*

Because the data deviated significantly from the normal distribution we have run Spearman's rank correlation test, which is also robust regarding the outliers. As we described in “2.6. Correlation between the abundance of miRNAs and the number of miRNA targets” section of Methods we obtained statistically significant correlation with Spearman's rank correlation coefficient rho = 0.52 between target counts and mature miRNA read counts and rho = 0.67 between target counts and stem-loop transcripts read counts (*p*-value < 2.2e-16 in both cases). We provided the plots on logarithmic scale.

We also tested correlation between schizophrenia miRNA target counts and read counts for these miRNAs and demonstrated statistically significant correlation with Spearman's coefficient, rho = 0.47 (*p*-value = 1.012e-09) for mature schizophrenia-related miRNA read counts; and rho = 0.49 (*p*-value = 2.255e-11) for SZ miRNAs stem loops.

*11. On differential methylation of repetitive elements, the authors write: “We studied the methylation of repetitive elements by comparing the sequences of the differentially methylated probes in all three data sets in* [6] *to Repbase, a collection of all repetitive elements in eukaryotic genomes* [35]*. While we did not find any particular repetitive element enriched in the differentially methylated probes, using Student’s t-test we did find data sets 1 and 2 significantly more methylated (p-value < 1e-5) if they matched a repetitive element when compared to the methylation value distributions derived from the entire sets (Fig.*5*a and b) whereas for data set 3 the repetitive element matching probes were slightly less methylated than the entire set (p-value = 0.017).” This section is not clear to this reviewer, while the results seem inconsistent. This section should either be elaborated or deleted.*

We expanded this section, we hope it is clear now. In principle, we compared the sequences of each probe (that was found differentially methylated in the Wockner paper) to each repetitive element in Repbase. When we compare the methylation value distributions for the repetitive probes only (in Fig. 5b) to the distributions of the methylation values of all differentially methylated probes (Fig. 5a), it is clear that for set 1 and set 2 (x1 and x2 in Fig. 5) the patients’ have higher methylations levels (the positive side of the histograms in Fig. 5b increase when compared to Fig. 5a) whereas for the x3 data set (also from the Wockner set, comparing two patient subgroups) methylation distribution (histogram) remains the same for the repetitive subset (in Fig. 5b) when compared to the total set (to be more precise, it is marginally different, *p*-value being 0.017).

*12. In their Conclusions section the authors only reiterate earlier findings that are not the topic of the current manuscript. In summary, this paper presents a number of very interesting findings, but the work needs to be elaborated and the results should be placed in a biological/evolutionary context.*

We changed the Conclusions section accordingly and expanded on the biological meaning in the Discussion. Thanks for the suggestions and the overall positive impression about our findings.

### Responses to Reviewer 2 (Sandor Pongor)

*In this work the authors analyzed several recent data sets: (i) a methylome study, (ii) microRNAs’ experimentally verified targets collected from the literature, (iii) STRING, a protein-protein interaction database, (iv) Genecards, (v) regulatory regions of human genes and transcription factor binding sites mapped to the human genome, concluding that GABBR1 plays a significant role in the etiology of schizophrenia. It is certainly of interest, considering that schizophrenia affects 1 % of the population with heavy toll on society both in financial and human terms. Interestingly, the work is in line with a previous finding by the authors, also published in Biology Direct, where they also concluded that the downregulation of GABBR1 via an endogenous retroviral element might be the cause of schizophrenia. In this study they start their investigation with microRNAs implicated in schizophrenia, finding that microRNA targets form a scale-free network and, accordingly, the top ten microRNAs regulate 80 % of all schizophrenia-related genes. The top two microRNAs regulate 40-52 % of all genes, the ratio depending on the data set they use. They suggest that the more relevant the gene set is to schizophrenia, the higher this ratio is, highlighting the importance of microRNAs in schizophrenia. The top two microRNAs both regulate GABBR1, from which they conclude again that this gene is of special interest in schizophrenia.*

Thanks for the overall positive view.

*The authors may want to deal with the following issues: 1. The selection of genes in* Additional file 6: *Table S2 seems rather arbitrary. While they also implicate AKT1 as a gene of importance from the protein-protein interaction network they draw of genes regulated by one of the top 2 microRNAs, there is no data about transcription factor binding sites (TFBSs) for AKT1.*

We tried to select the most important genes. There was no available data on the cis regulatory and promoter regions in the Thurman data set about AKT1.

*2. There is no data for KCNJ9 (in the same table) whereas it directly interacts with GABBR1.*

We fixed this, the gene is now included in Additional file 4: Table S2.

*3. It is difficult to see the significance of the TFBSs. The authors should at least calculate the correlation between the genes they list in Additional file**6**: Table S2.*

We felt that this would place too much emphasis on the TFBSs and draw the attention away from the main focus of the paper, which was the microRNA-regulation and protein interaction networks.

*4. Does the - approximately - scale-free nature of the network have a biological significance?*

See our response in this respect to reviewer 1 above. It is the robustness of the network that is supported by the scale-free nature of the network, which will probably increase over time as more microRNA-target relations will get discovered, considering how novel and intensely researched the subject is.

### Responses to Reviewer 3 (Zoltan Gaspari)

*Schizophrenia is a disease with unknown aetiology despite numerous efforts to find its cause for over more than a century. In this study Gumerov and Hegyi approach the subject from the point of view of microRNAs. They combine microRNA target data with a methylation study conducted in schizophrenic brains, a protein-protein interaction database, STRING, and various gene sets such as Genecards and Malacards, mostly derived from text mining of the literature. The authors find that the mostly hypermethylated gene in the methylation study, GABBR1 is also the target of the top two microRNAs with the highest number of targets in their set. Combining this with protein-protein interaction data they find that most proteins form a network of interactions with two hubs where one of the hubs is again GABBR1 while the other hub is AKT1, protein kinase B, an important protein in signal transduction. From this they conclude that GABBR1 might play a causative role in schizophrenia. I think the work is original and describes observations that can be of importance in understanding schizophrenia.*

Thanks for taking on our manuscript and for the positive tone.

*I have a few notes that I ask the authors to address in the final published version:*

*1. If the genes in question form a network by being targeted by shared microRNAs as they suggest, should not these microRNAs also be hyper- or hypomethylated in the methylation study they analyze in this study? There is no mentioning of this in the manuscript.*

There were only a handful of differentially methylated microRNAs in the methylome study we based our study on, none of them particularly outstanding.

*2. How do the transcription factors with binding sites in the cis regions of the genes in the network in Fig.*3*relate to the shared microRNAs? Aren’t they more important in the regulation of the interacting genes than the microRNAs they focus on? (Also, there is no data on promoter transcription factor binding sites, only on cis regulatory elements). This gives an incomplete picture.*

We found that the two different types of regulatory mechanisms (transcription factor binding sites in the cis regions and microRNA-targeting) are two unrelated mechanisms, which we mention in the paper. We wanted to focus on the latter (microRNAs – protein targets) in this study.

*3. There seems to be an inconsistency regarding the hyper- and hypomethylation of the genes that interact with each other. E.g. the authors claim that AKT1 is downregulated in schizophrenics whereas AKT1 seems to be only mildly hypermethylated based on its shade of grey in Fig.*3*.)*

This is a very good point. However, we found that most genes are both hyper- and hypomethylated in the Wockner study. Also, the difference between earlier studies and the current methylome study may lie in the fact that while the study that found AKT1 consistently downregulated in schizophrenic patients investigated the gene expression patterns in the blood of patients and with recent-onset disease, the methylome study analyzed the brains of (dead) schizophrenic patients, with apparently longer histories of the disease.

*4. It would be interesting to see whether in the full available interaction network of the proteins in question (i.e. not only those regulated by the selected miRNAs and/or indicated in schizophrenia) GABBR1 and AKT1 still stand out with some properties.*

We tried to generate a network using PPIs in STRING for the fully available interaction network. However, AKT1 seems to have a lot more (known) interactions than GABBR1, therefore the picture seemed a lot more complex and nonspecific. As far as we know, there is no PPI database that would show only tissue-specific protein interactions.

*Minor issues: − When using data from mirbase.org, were the tissue origins of the miRNAs considered? –*

Yes, we did additional plots, as there was available data for the prefrontal cortex only. Our findings are still valid (Additional file 5: Figure S3).

*In Fig.*4*, might the existence of the correlation be better visualized on a logarithmic scale? –*

Yes, thanks for the suggestion, we did it and it looks better.

*In the abstract the authors mention a scale-free network. I would refrain from this notation even if the power-law distribution for miRNA targets is valid because the nodes in the full network are not equivalent (miRNAs and regulated proteins).*

We expanded on this issue clarifying the networks we had in mind and removed the term “scale-free network”. Using the known miRNA-target relations we constructed two types of networks: a miRNA- > target and a miRNA-miRNA network. The network of miRNAs and the genes regulated by them, not including interactions between these genes, is a bipartite directed network with miRNA- > target directed links. This network can be characterized by in-degree (in this case the number of miRNAs controlling a given gene) distribution and out-degree (the number of genes controlled by a given miRNA) distribution. In Fig. 2 we depicted the out-degree distribution of this network. We tested goodness of fit using Kolmogorov-Smirnov statistic as described in Methods, which demonstrated that this distribution follows the power law.

Supplementary information is available at the journal’s website.

## Additional files

Additional file 1: Figure S1. Pairwise overlaps among the three datasets: A – cross-tabulation of verified schizophrenia-related miRNA targets (SZmiRNATargets) with differentially methylated genes from the subset 2 of [6] (WocknerTab2); B – cross-tabulation of intersection A with schizophrenia genes in Genecards; C – cross-tabulation of intersection A with schizophrenia genes in Malacards. (DOC 211 kb) Additional file 2: Table S1. All experimentally verified targets that were found differentially methylated in schizophrenics by Wockner et al. [6] (in promoters overlapping a CpG island) of all microRNAs associated with schizophrenia. The number of promoter and cis elements associated with each gene as found by Thurman et al. [40] are shown in columns 2 and 3, respectively. (XLSB 247 kb) Additional file 3: Figure S2. Proportions of genes regulated by schizophrenia miRNAs in 2 datasets. A – differentially methylated genes from the subset 2 of [6]; B – intersection of differentially methylated genes in the subset 2 of [6] with schizophrenia genes in Genecards. X axis – ranks of miRNAs according to the number of regulated genes. First 10 miRNAs: miR-335-5p, miR-26b-5p, miR-16-5p, miR-124-3p, miR-92a-3p, miR-484, miR-155-5p, let-7b-5p, miR-193b-3p, miR-21-5p. (DOC 139 kb) Additional file 4: Table S3. Results of randomization and statistical tests for top 10 schizophrenia miRNAs. Columns from the left to the right: 1 - miRNA, 2 - number of genes regulated by miRNA in the subset of genes studied in this paper, 3- maximum number of connections in the undirected graph with the number of nodes equal to the number of genes regulated by a given miRNA, 4 – actual number of connections in this graph, 5 – normalized number of connections in the graph, 6 – average number of connections in 3000 simulated graphs with the number of nodes equal to the number of genes regulated by a given miRNA, constructed based on the randomly selected schizophrenia genes from Genecards, 7 – normalized average number of connections in these 3000 graphs, 8 – *p*-values of Wilcoxon signed-rank tests. (DOC 40 kb) Additional file 5: Figure S3. Scatterplot of the number of experimentally validated targets (taken from mirTarBase) vs. the abundance of miRNAs (from mirbase.org) implicated in schizophrenia. A and B – mature miRNA read counts and stem-loop transcripts read counts averaged across several tissues presented in mirbase.org; C and D – mature miRNA read counts and stem-loop transcripts read counts of miRNAs expressed in frontal cortex. (DOC 301 kb) Additional file 6: Table S2. The number of different transcription factor-binding sites for 15 GABBR1-related genes (binding partners and neighborhood genes of GABBR1 in Fig. 3) mapped to the cis regulatory elements as identified by Thurman et al. [40] based on the compilation of all TFBSs in the human genome by [41]. (XLSB 58 kb)

## Abbreviations

GABBR1: GABA (gamma-aminobutyric acid) receptor B1
AKT1: Protein kinase B
SNP: Single nucleotide polymorphism
MHC region: Major histocompatibility region
PPI: Protein-protein interactions
ATF2: Activating transcription factor 2
miRNA: microRNA
NPY: Neuropeptide Y
PMI: Postmortem interval
