# Supplementary material for: MicroRNA-derived network analysis of differentially methylated genes in schizophrenia, implicating GABA receptor B1 [GABBR1] and protein kinase B [AKT1]
Source: Biol Direct. 2015 Oct 8;10:59. doi: 10.1186/s13062-015-0089-y (PMC4598960; doi:10.1186/s13062-015-0089-y)
Supplement: Additional file 1: Figure S1. — Pairwise overlaps among the three datasets: A – cross-tabulation of verified schizophrenia-related miRNA targets (SZmiRNATargets) with differentially methylated genes from the subset 2 of [6] (WocknerTab2); B – cross-tabulation of intersection A with schizophrenia genes in Genecards; C – cross-tabulation of intersection A with schizophrenia genes in Malacards. (DOC 211 kb) [file 13062_2015_89_MOESM1_ESM.doc]

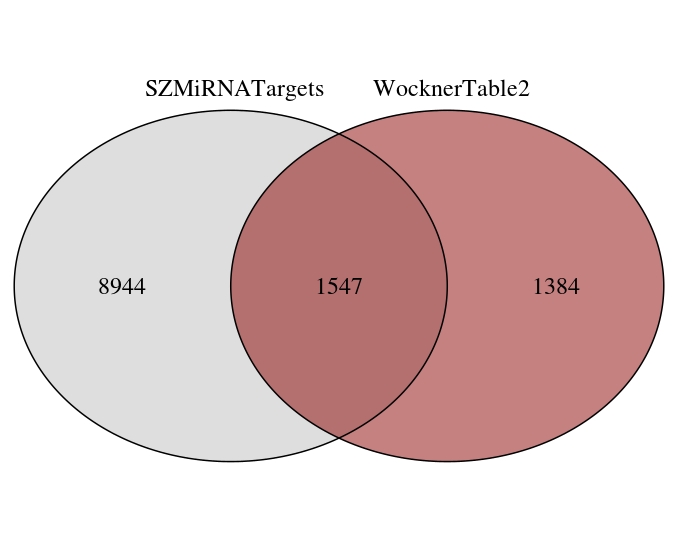


A.


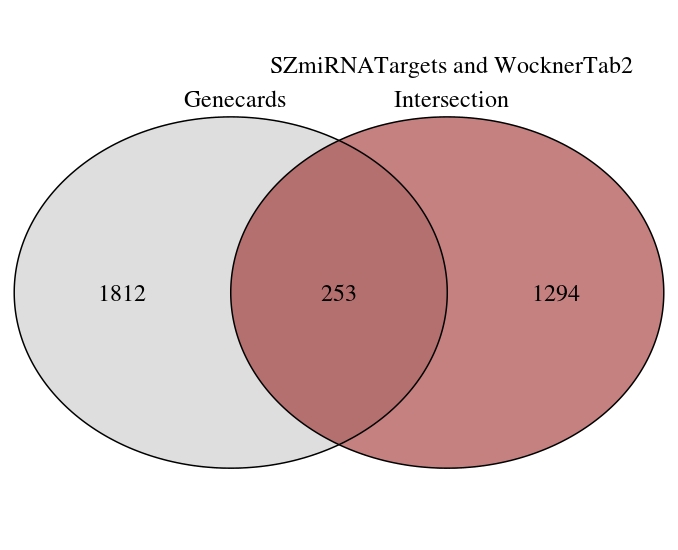
 B. C.


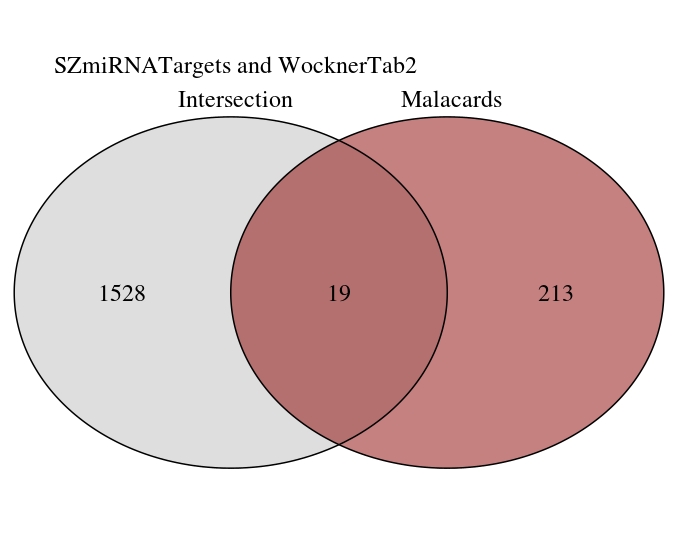


**Additional file 1: Figure S1**. Pairwise overlaps among the three datasets: **A** – intersection between verified schizophrenia-related miRNA targets (SZmiRNATargets) and differentially methylated genes in the subset 2 of (1) (WocknerTab2); **B** – overlap between intersection A and schizophrenia genes in Genecards; **C** – overlap between intersection A and schizophrenia genes in Malacards.
