# Supplementary material for: MicroRNA-derived network analysis of differentially methylated genes in schizophrenia, implicating GABA receptor B1 [GABBR1] and protein kinase B [AKT1]
Source: Biol Direct. 2015 Oct 8;10:59. doi: 10.1186/s13062-015-0089-y (PMC4598960; doi:10.1186/s13062-015-0089-y)
Supplement: Additional file 3: Figure S2. — Proportions of genes regulated by schizophrenia miRNAs in 2 datasets. A – differentially methylated genes from the subset 2 of [6]; B – intersection of differentially methylated genes in the subset 2 of [6] with schizophrenia genes in Genecards. X axis – ranks of miRNAs according to the number of regulated genes. First 10 miRNAs: miR-335-5p, miR-26b-5p, miR-16-5p, miR-124-3p, miR-92a-3p, miR-484, miR-155-5p, let-7b-5p, miR-193b-3p, miR-21-5p. (DOC 139 kb) [file 13062_2015_89_MOESM3_ESM.doc]

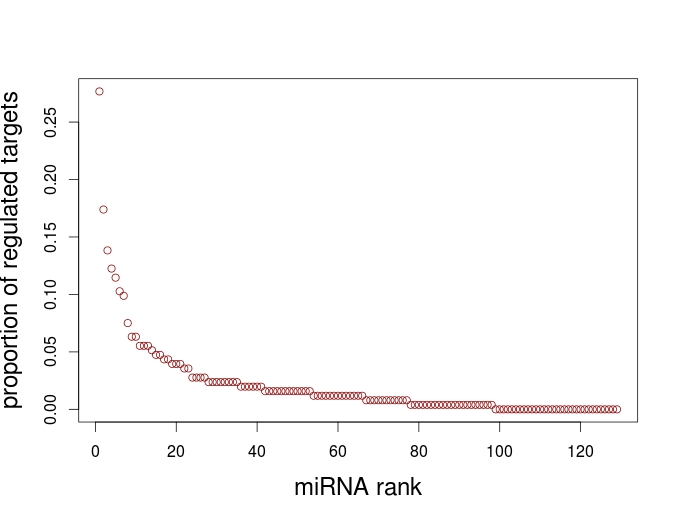


A. B.


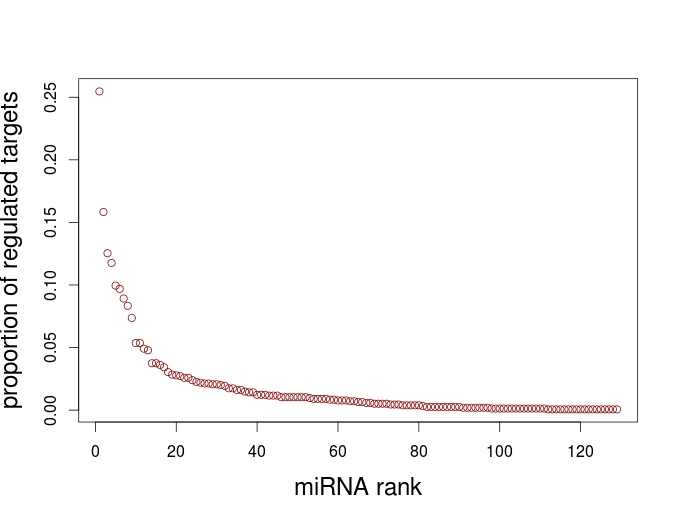


**Additional file 3 Figure S2**. Proportions of genes regulated by schizophrenia miRNAs in 2 datasets.

**A** – differentially methylated genes from the subset 2 of (1); **B** – intersection of differentially methylated genes in the subset 2 of (1) with schizophrenia genes in Genecards. X axis – ranks of miRNAs according to the number of regulated genes. First 10 miRNAs: miR-335-5p, miR-26b-5p, miR-16-5p, miR-124-3p, miR-92a-3p, miR-484, miR-155-5p, let-7b-5p, miR-193b-3p, miR-21-5p.
