# Supplementary material for: MicroRNA-derived network analysis of differentially methylated genes in schizophrenia, implicating GABA receptor B1 [GABBR1] and protein kinase B [AKT1]
Source: Biol Direct. 2015 Oct 8;10:59. doi: 10.1186/s13062-015-0089-y (PMC4598960; doi:10.1186/s13062-015-0089-y)
Supplement: Additional file 4: Table S3. — Results of randomization and statistical tests for top 10 schizophrenia miRNAs. Columns from the left to the right: 1 - miRNA, 2 - number of genes regulated by miRNA in the subset of genes studied in this paper, 3- maximum number of connections in the undirected graph with the number of nodes equal to the number of genes regulated by a given miRNA, 4 – actual number of connections in this graph, 5 – normalized number of connections in the graph, 6 – average number of connections in 3000 simulated graphs with the number of nodes equal to the number of genes regulated by a given miRNA, constructed based on the randomly selected schizophrenia genes from Genecards, 7 – normalized average number of connections in these 3000 graphs, 8 – p-values of Wilcoxon signed-rank tests. (DOC 40 kb) [file 13062_2015_89_MOESM4_ESM.doc]

**Additional file 4 Table S3**. Results of randomization and statistical tests for top 10 schizophrenia miRNAs. Columns from the left to the right: 1 – miRNA; 2 - number of genes regulated by miRNA in the subset of genes studied in this paper; 3 – maximum number of connections in the undirected graph with the number of nodes equal to the number of genes regulated by a given miRNA; 4 – actual number of connections in this graph; 5 – normalized number of connections in the graph; 6 – average number of connections in 3000 simulated graphs with the number of nodes equal to the number of genes regulated by a given miRNA, constructed based on the randomly selected schizophrenia genes from Genecards; 7 – normalized average number of connections in these 3000 graphs; 8 – p-values of Wilcoxon signed-rank tests.

| miRNA | Number of targets | Max  Connections | Actual  Connections | Normalized actual  Connections | Mean  Connections  In3000SimSet | Normalized  Mean Connections  In3000SimSet | p-val |
| --- | --- | --- | --- | --- | --- | --- | --- |
| hsa-miR-335-5p | 308 | 47278 | 649 | 0.01373 | 510.6 | 0.0108 | 0 |
| hsa-miR-26b-5p | 188 | 17578 | 286 | 0.01627 | 188.6 | 0.01073 | 0 |
| hsa-miR-16-5p | 168 | 14028 | 323 | 0.02303 | 151.8 | 0.01082 | 0 |
| hsa-miR-124-3p | 143 | 10153 | 231 | 0.02275 | 109 | 0.01073 | 0 |
| hsa-miR-92a-3p | 126 | 7875 | 162 | 0.02057 | 85.1 | 0.01081 | 0 |
| hsa-miR-484 | 118 | 6903 | 152 | 0.02202 | 74.6 | 0.01081 | 0 |
| hsa-miR-155-5p | 107 | 5671 | 112 | 0.01975 | 61.1 | 0.01077 | 0 |
| hsa-let-7b-5p | 104 | 5356 | 121 | 0.02259 | 57.3 | 0.01069 | 0 |
| hsa-miR-193b-3p | 95 | 4465 | 115 | 0.02576 | 48.6 | 0.01089 | 0 |
| hsa-miR-21-5p | 78 | 3003 | 100 | 0.0333 | 32.1 | 0.0107 | 0 |

**References**

1. Wockner LF, Noble EP, Lawford BR, Young RM, Morris CP, Whitehall VL, et al. Genome-wide DNA methylation analysis of human brain tissue from schizophrenia patients. Translational psychiatry. 2014;4:e339.
