# Supplementary material for: MicroRNA-derived network analysis of differentially methylated genes in schizophrenia, implicating GABA receptor B1 [GABBR1] and protein kinase B [AKT1]
Source: Biol Direct. 2015 Oct 8;10:59. doi: 10.1186/s13062-015-0089-y (PMC4598960; doi:10.1186/s13062-015-0089-y)
Supplement: Additional file 5: Figure S3. — Scatterplot of the number of experimentally validated targets (taken from mirTarBase) vs. the abundance of miRNAs (from mirbase.org) implicated in schizophrenia. A and B – mature miRNA read counts and stem-loop transcripts read counts averaged across several tissues presented in mirbase.org; C and D – mature miRNA read counts and stem-loop transcripts read counts of miRNAs expressed in frontal cortex. (DOC 301 kb) [file 13062_2015_89_MOESM5_ESM.doc]

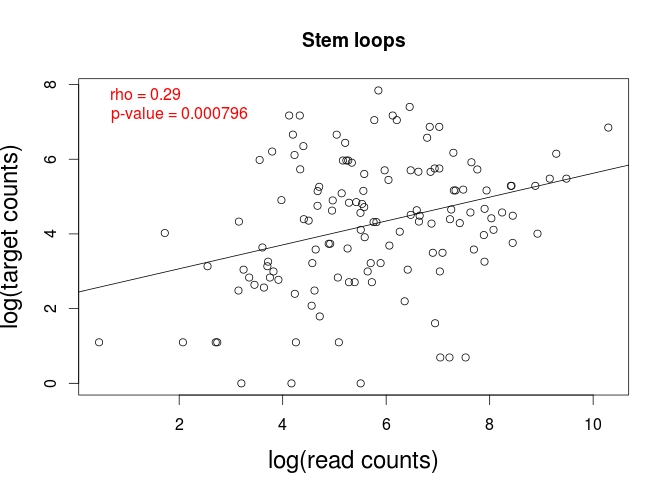

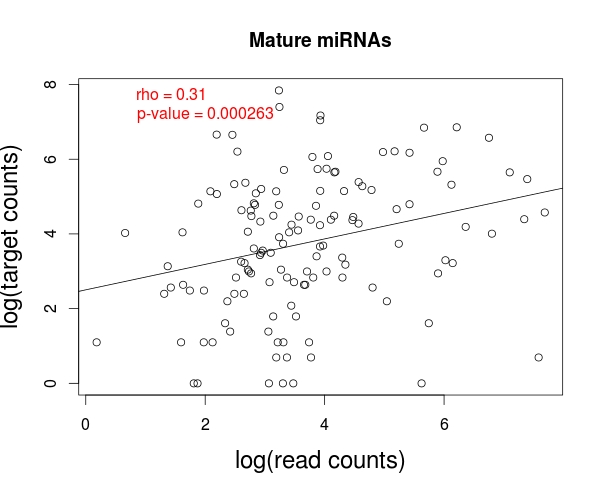
 A. B.


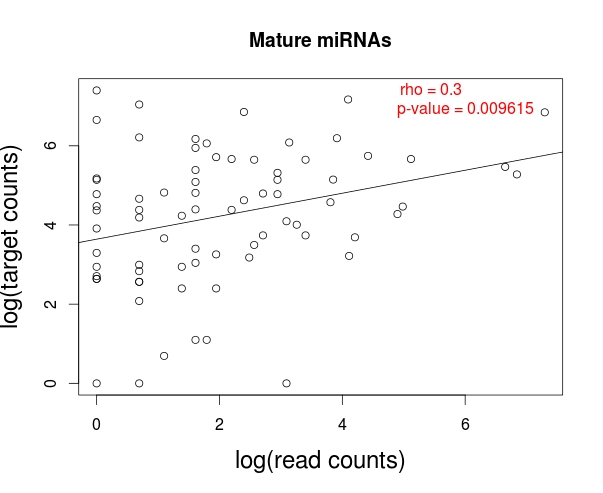

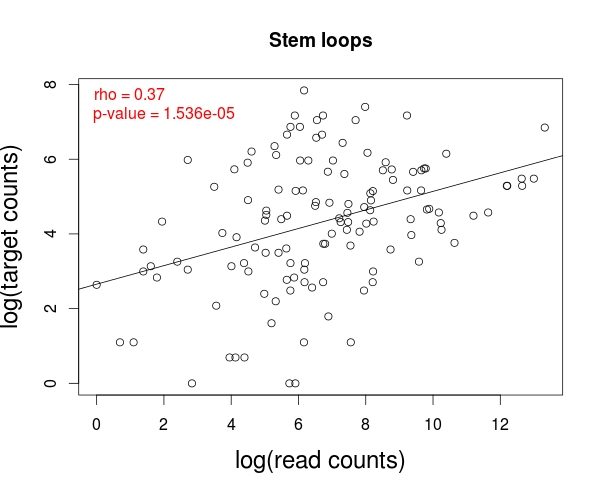
 C. D.

**Additional file 5 Figure S3**. Scatterplot of the number of experimentally validated targets (taken from mirTarBase) vs. the abundance of miRNAs (from mirbase.org) implicated in schizophrenia. **A** and **B** – mature miRNA read counts and stem-loop transcripts read counts averaged across several tissues presented in mirbase.org; **C** and **D** – mature miRNA read counts and stem-loop transcripts read counts of miRNAs expressed in frontal cortex.
